# The Novel Antioxidant Compound JSH-23 Prevents Osteolysis by Scavenging ROS During Both Osteoclastogenesis and Osteoblastogenesis

**DOI:** 10.3389/fphar.2021.734774

**Published:** 2021-09-09

**Authors:** Liangwei Mei, Yi Zheng, Teng Ma, Bing Xia, Xue Gao, Yiming Hao, Zhuojing Luo, Jinghui Huang

**Affiliations:** ^1^ Department of Orthopaedics, Xijing Hospital, the Fourth Military Medical University, Xi’an, China; ^2^ Faculty of Life Sciences, Northwest University, Xi’an, China

**Keywords:** JSH-23 (PubChem CID: 16760588), osteoclast (OC), osteoblast (OB), ROS–reactive oxygen species, Nrf2, HO-1 (heme oxygenase-1)

## Abstract

Inflammatory osteolysis is a pathological skeletal disease associated with not only the production of inflammatory cytokines but also local oxidative status. Excessive reactive oxygen species (ROS) promote bone resorption by osteoclasts and induce the apoptosis of osteoblasts. In consideration of the lack of effective preventive or treatments options against osteolysis, the exploitation of novel pharmacological compounds/agents is critically required. In our study, we found that a novel antioxidant compound, JSH-23, plays a role in restoring bone homeostasis by scavenging intracellular ROS during both osteoclastogenesis and osteoblastogenesis. Mechanically, JSH-23 suppressed RANKL-induced osteoclastogenesis, bone resorption and the expression of specific genes (including NFATc1, c-Fos, TRAP, CTSK and DC-STAMP) via inhibition of the NF-κB signaling pathway. Meanwhile, JSH-23 suppressed RANKL-induced ROS generation via the TRAF6/Rac1/NOX1 pathway and the enhanced expression of Nrf2/HO-1. In addition, JSH-23 attenuated H_2_O_2_-induced apoptosis and mineralization reduction in osteoblasts by reducing ROS production and enhancing Nrf2/HO-1 expression. Our *in vivo* results further revealed that JSH-23 exerts its protective effects on bone mass through its antioxidant activity. In conclusion, our results show that the application of JSH-23 might be a novel and plausible strategy for the treatment of osteolysis-related disease.

## Introduction

Inflammatory osteolytic diseases, including rheumatoid arthritis, periodontitis and osteomyelitis, is characterized by an imbalance in bone remodeling triggered by an increased number of osteoclasts in conjunction with a decreased in the bone formation ability of osteoblasts, ultimately leading to pathological bone destruction (Jimi et al., 2004; Smolen et al., 2016; Wang et al., 2021). Local inflammation in the bone induces the production of pro-osteoclastogenic cytokines, including tumor necrosis factor-α (TNF-α) and interleukins (ILs), which promote the differentiation and activation of bone-resorbing osteoclasts by increasing the expression of receptor activator of nuclear factor κB ligand (RANKL)(Boyle et al., 2003; Boutet et al., 2017). Current pharmacological interventions, including estrogen replacements, bisphosphonates and anti-RANKL antibody (denosumab), exert somewhat beneficial effects against osteoclast-mediated osteolysis (McClung et al., 2006; Zhou et al., 2020). However, serious undesirable side effects, including cardiovascular events, nephrotoxicity, osteonecrosis of the jaw, atypical fractures and gastrointestinal distress, are becoming increasingly prominent, limiting their long-term use (Kotian et al., 2016; Patel et al., 2018).

Many recent studies have shown that local inflammation further induces the production of reactive oxygen species (ROS), which play a dual role in bone homeostasis (El-Benna et al., 2016; Tao et al., 2020). Under physiological conditions, ROS are essential for bone remodeling. However, under pathological conditions such as those in inflammatory arthritis and estrogen-deficient osteoporosis, the overproduction of ROS is often linked with excessive bone loss (Chen et al., 2019). At the cellular level, ROS represent a double-edged sword. During RANKL-induced osteoclast formation, ROS maintain their normal functions in differentiation, survival, activation and bone resorption (Ashtar et al., 2020). However, they can reduce osteoblast formation by inducing osteoblast apoptosis and decreasing osteoblast activity (Han et al., 2019). Therefore, different routes can lead to the same endpoint, and imbalance attributable to excess bone resorption compared with bone formation leads to osteolysis. Thus, limiting the excessive production of intracellular ROS has been assumed to prevent the extreme formation of osteoclasts and apoptosis of osteoblasts induced by local inflammation (Bodega et al., 2019; Tao et al., 2020).

Extensive studies have examined the effects of various antioxidants on osteolytic diseases (Horcajada and Offord, 2012; Abuohashish et al., 2013). For example, pseurotin A and loureirin B inhibit osteoclastogenesis by attenuating ROS activities but have no effect on osteoblast differentiation (Chen et al., 2019; Liu et al., 2019). In addition, tabersonine, Fufang Lurong Jiangu capsule and Z-guggulsterone promote antioxidant pathways to resist apoptosis in osteoblastic cells, but their effects on osteoclasts are unknown (Xu et al., 2019; Jin et al., 2020; Sun et al., 2020). Because both osteoblasts and osteoclasts are involved in the pathological process of osteolysis, these compounds affect only one type of bone cell, which limits their clinical translational application. Under these circumstances, it is very necessary to find novel antioxidant compounds that target both osteoclasts and osteoblasts to treat osteolytic diseases.

JSH-23 is an NF-κB inhibitor that inhibits NF-κB transcriptional activity without affecting IκB degradation. Previous study suggested that JSH-23 has an anti-inflammatory effect caused by its regulation of lipopolysaccharide (LPS)-induced nuclear NF-κB p65 translocation (Shin et al., 2004; Arias-Salvatierra et al., 2011), and JSH-23 was found to regulate the antioxidant defense machinery in depressive-like behaviors and diabetic neuropathy (Kumar et al., 2011; Wang et al., 2018); furthermore, JSH-23 was shown to inhibit *Staphylococcus aureus* or *Staphylococcus aureus* protein A-induced osteoclastogenesis (Ren et al., 2017). As bone homeostasis involves the balance between bone resorption by osteoclasts and bone formation by osteoblasts, and ROS are known to play a key role in bone homeostasis, we hypothesized that JSH-23 plays a role in restoring bone homeostasis by scavenging intracellular ROS during both osteoclastogenesis and osteoblastogenesis, thereby restraining bone loss.

## Materials and Methods

### Reagents

Alpha minimum essential medium (α-MEM), penicillin/streptomycin, and fetal bovine serum (FBS) were purchased from Excell (Excell Bio, Shanghai, China). Cell culture plate was purchased from *In Vitro* Scientific (Hangzhou Xinyou Biotechnology Co., Ltd, China). T75 flask was purchased from Jet Biofil (Guangzhou, China). JSH-23 (purity = 99.07%, batch No. S735101, CAS No. 749886–87–1) was purchased from Selleck (Houston, TX, United States). A Cell Counting Kit-8 (CCK-8) assay kit was purchased from MultiSciences (Hangzhou, China). Primary antibodies against NFATc1 (ab177464), c-Fos (ab222699), p-p65 (ab76302), p65 (ab32536), p-Akt (ab192623), Akt (ab179463), p-ERK (ab201015), ERK (ab184699), p-JNK (ab124956), JNK (ab179461), p-p38 (ab178867), p38 (ab170099), TRAF6 (ab33915), Rac1 (ab180683), NOX1 (ab131088), HO-1 (ab68477), Catalase (ab209211), NQO1 (ab80588), GSR (ab124995), Bcl-2 (ab182858), Bax (ab182733), Runx2 (ab192256), BMP2 (ab214821), and GAPDH (ab8245) were purchased from Abcam (Cambridge, MA, United States). Primary antibody against OCN (AB10911) was purchased from Sigma-Aldrich (St. Louis, MO). Primary antibody against p-IκBα (#2859), IκBα (#4814), Cleaved Caspase9 (#9507), Cleaved Caspase3 (#9664), and Nrf2 (#12721) were purchased from Cell Signaling Technology (Danvers, MA, United States). Recombinant mouse M-CSF and mouse RANKL were purchased from R&D Systems (Minneapolis, MN, United States). RNA interference sequences were purchased from GeneChem (Shanghai, China).

### Cell Culture

Bone marrow macrophages (BMMs) were used as osteoclast precursors and isolated from the bone marrow of four-week-old C57BL/6 mice that were euthanized according to procedures approved by the Fourth Military Medical University. The BMMs were grown in α-MEM supplemented with 10% FBS, 1% penicillin-streptomycin solution, 30 ng/ml M-CSF and incubated in an atmosphere of 5% CO_2_ at 37°C. The culture medium was changed every 2 days.

MC3T3-E1 cells were used in our experiments as osteoblasts. The cells were cultured in α-MEM supplemented with 10% FBS and 1% penicillin-streptomycin solution in an atmosphere of 5% CO_2_ at 37°C. The culture medium was changed every 2 days.

### Cell Viability Assay

BMMs were seeded into 96-well plates (8 × 10^3^ cells/well) and incubated at 37°C. After 24 h, the cells were treated with various concentrations of JSH-23 (range 1–200 μM) and cultured for 48 or 96 h. Ten microliters of CCK-8 buffer was added to each well, the cells were incubated at 37°C for 1 h, and the absorbance was measured at 450 nm on an ELX800 microplate reader (Bio-Tek, Vermont).

### *In Vitro* Osteoclastogenesis and F-Actin Ring Immunofluorescence Assay

BMMs were seeded at a density of 8 × 10^3^ cells/well in 96-well plates and treated in complete medium containing 30 ng/ml M-CSF and 100 ng/ml RANKL with or without different concentrations of JSH-23 (10, 20 and 40 μM). After 5 days, the cultured cells were fixed with 0.25% glutaraldehyde for 20 min at room temperature and then washed three times with PBS. The tartrate-resistant acid phosphatase (TRAP) enzymatic activity of the cells was detected using a leukocyte acid phosphatase staining kit according to the manufacturer’s procedures. TRAP-positive cells (>3 nuclei) were regarded as osteoclasts and counted under a light microscope (Nikon Corporation, Tokyo, Japan). After TRAP staining, the cells were washed three times with PBS and stained with Actistain 488 Fluorescent Phalloidin (Cytoskeleton, Denver, CO, United States) at room temperature for 30 min in the dark. Then BMMs were washed three times with PBS, and the nuclei were visualized with 1 mg/ml DAPI. Images of osteoclastic rings were obtained using an immunofluorescence microscope.

### Bone Resorption Assay

After RANKL stimulation for 3 days, equal numbers of BMM-derived preosteoclasts were seeded onto bovine bone slices and treated with JSH-23 (10, 20 and 40 μM) for another 48 h. Cells that had adhered to the bone slices were then removed by mechanical agitation and sonication. Resorption pits were visualized under an SEM (FEI Quanta 250), and the bone resorption area was quantified using ImageJ software.

### Quantitative Real-Time PCR (QPCR)

Cells from different treatment groups were washed with cold PBS and lysed with RNAiso Plus (Takara Bio, Otsu, Japan) to obtain RNA according to the manufacturer’s protocol. The total RNA was then reverse-transcribed to obtain cDNA. The cDNA was used for quantitative real-time PCR, which was performed with a CFX96 Real-Time PCR Detection System (Bio-Rad, Hercules, CA, United States). The values were normalized to the levels of GAPDH. The primer sequences (Sino Biological Inc., Beijing, China) are listed in Supplementary Table S1.

### Western Blotting

Cultured cells from different treatment groups were washed with cold PBS three times for 5 min. RIPA lysis buffer (Sangon Biotech) was used to lyse the cells for 20 min at 4 C. Then, the obtained cell lysates were centrifuged at 10,000 × *g* for 10 min. The obtained supernatants were collected and dissolved in loading buffer. A 10 μL mixture was separated on 10% SDS-PAGE gels, and the separated proteins were then transferred to PVDF membranes (Bio-Rad, Hercules, CA, United States). The membranes were washed with Tris-buffered saline-Tween 20 (TBST) twice for 10 min and blocked with 5% nonfat dry milk at room temperature for 1 h. Next, the membranes were incubated with primary antibodies overnight at 4°C and with secondary antibodies for 1 h at room temperature. The protein bands were detected using electrochemical luminescence reagent (Millipore, Billerica, MA, United States). The grayscale values of the bands were quantified using ImageJ software (National Institutes of Health, Bethesda, MD, United States).

### Detection of ROS Production

2′,7′-Dichlorodihydrofluorescein diacetate (DCFH-DA) staining was performed to measure ROS production *in vitro*. Cells were fixed in 4% formaldehyde for 15 min and then washed with PBS. DCFH-DA (10 μM) was added to the wells, and the cells were incubated for 30 min at 37°C. The cells were washed twice with PBS, and fluorescence images were obtained. The fluorescence intensity was quantified.

*In vivo* ROS production was assessed by injection of dihydroethidium (DHE) into the calvaria 24 h prior to killing, according to a protocol previously reported (Ni et al., 2020).

### Immunofluorescence Staining

Cells were washed with PBS three times, fixed in 4% paraformaldehyde for 30 min at room temperature, blocked with 5% (w/v) BSA in PBST, and immunostained with primary antibodies overnight at 4°C followed by secondary antibodies. After being washed with PBS three times, the cells were stained with DAPI and observed under a fluorescence microscope.

### Cell Transfection

BMMs or MC3T3-E1 cells were transfected with siRNA using Lipofectamine 3,000 (Invitrogen) according to the manufacturer’s protocol. Briefly, cells were seeded in 6-well plates at a density of 2 × 10^4^ cells/well and transfected with 20 nM siRNA. After 6 h, the medium was replaced with complete α-MEM medium, and the cells were cultured for another 48 h. The efficiency of transfection was observed under a fluorescence microscope. Cells were analyzed by western blotting to assess the expression of nuclear factor E2-related factor 2 (Nrf2) and heme oxygenase 1 (HO-1).

### Flow Cytometric Analysis of Apoptosis

After different treatments, MC3T3-E1 cells were washed with PBS and then labeled with FITC-Annexin V and PI in binding buffer according to the manufacturer’s instructions. Ten thousand events were recorded and analyzed to obtain the percentage of apoptotic cells using a FACScan flow cytometer (BD Bioscience, San Jose, CA, United States).

### Alkaline Phosphatase (ALP) Activity and Mineralization Analysis

ALP activity is expressed as a percentage of enzyme activity relative to the control value. Alizarin red (AR) staining was performed to measure the degree of mineralization. MC3T3-E1 cells were pretreated with JSH-23 for 24 h before induction of differentiation for seven or 21 days and were then exposed to 400 μM H_2_O_2_ for 4 h for detection of ALP and AR staining, respectively. The immobilized cells were treated with 5-bromo-4-chloro-3-indolyl phosphate/nitro blue tetrazolium (BCIP/NBT, 7 days) or with 2% AR solution (21 days) for 30 min at room temperature. Then, the cells were washed three times with PBS and observed under a light microscope.

### LPS-Induced Murine Calvarial Osteolysis Model *in vivo*

The animal experiments were approved by The Fourth Military Medical University. Twenty-four six-week-old female C57BL/6 mice were equally divided into four groups: the sham group (treated with PBS), the vehicle group (treated with LPS), the low group (treated with LPS and 1 mg/kg JSH-23), and the high group (treated with LPS and 3 mg/kg JSH-23). The doses of JSH-23 used in the animal experiments was determined based on previous reports (Kumar et al., 2011; Wang et al., 2018). The mice were subcutaneously injected at the calvarial center with PBS or JSH-23 24 h before injection of LPS. Seven days after LPS injection, all mice were sacrificed, and the calvaria were dissected and fixed in 4% paraformaldehyde for micro-computed tomography (CT) and histological analysis.

### Micro-CT Scanning

Three-dimensional images of the whole calvaria were reconstructed using a high-resolution micro-CT scanner (Skyscan 1,176; Skyscan; Aartselaar, Belgium). Images were acquired using a 50 kV X-ray tube voltage, an 800 μA current, an isotropic pixel size of 14.4 μM (1,024 × 1,024 pixel image matrix) and a 0.75 mm thick aluminum filter for beam hardening. A square region of interest around the midline suture was chosen for further qualitative and quantitative analysis after reconstruction.

### Histological and Histomorphometric Analyses

The specimens were decalcified with 10% EDTA solution for 3 weeks and then embedded in paraffin. Histological sections were prepared and stained for hematoxylin and eosin (H&E) staining and TRAP activity assessment. The sections were then photographed under a microscope. Bone histomorphometric analyses were performed using BIOQUANT OSTEO histomorphometry software (BIOQUANT Image Analysis Corporation, Nashville, TN).

Immunofluorescence was performed to analyze Runx2, OCN, Nrf2 and HO-1 expression *in vivo*. Briefly, paraffin-embedded sections were deparaffinized and incubated in 1 mM EDTA (pH = 8.0) at 80°C for 15 min to retrieve the antigens. Then, the samples were blocked with 10% normal goat serum and incubated with primary antibodies. Secondary antibodies and DAPI were further applied, and the samples were observed under a fluorescence microscope. The fluorescence signal intensity was analyzed using ImageJ.

### Statistical Analysis

All experimental data are presented as the mean ± SD obtained from three experiments, and statistical significance was determined by Student’s t-test and one-way ANOVA. *p* < 0.05 was considered to indicate significance.

## Results

### JSH-23 Suppresses RANKL-Induced Osteoclastogenesis and Bone Resorption Without Cytotoxicity

**FIGURE 1:**
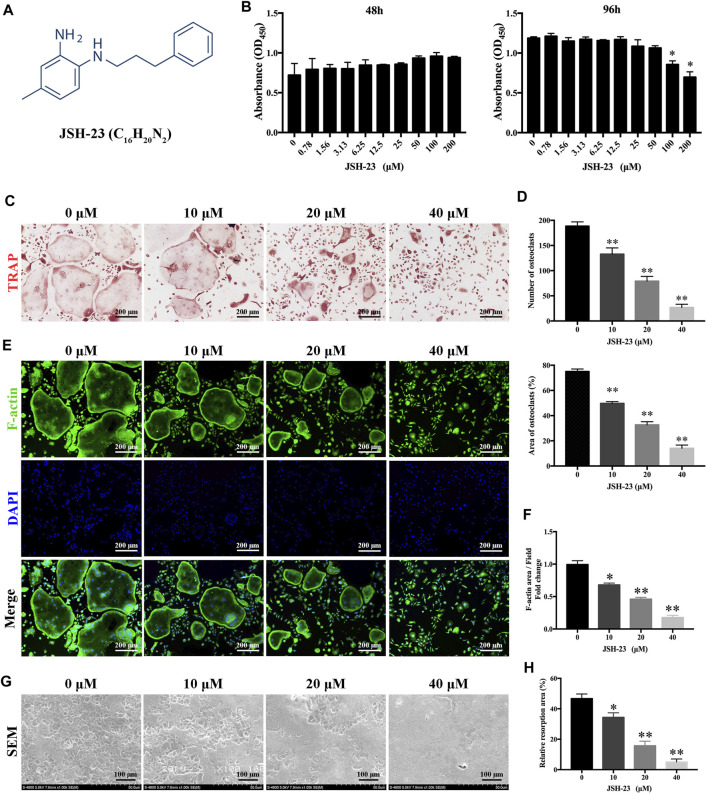
JSH-23 suppresses RANKL-induced osteoclastogenesis and bone resorption without cytotoxicity. **(A)** Chemical structure of JSH-23. **(B)** Effects of JSH-23 on BMMs viability as measured by CCK-8 assay. **(C)** BMMs were stimulated with M-CSF and RANKL in the presence of different concentrations of JSH-23 (10, 20, and 40 μM). TRAP staining showed that JSH-23 inhibited osteoclastogenesis dose-dependently. **(D)** Quantification of TRAP-positive cell numbers and area per well. **(E)** Cells were fixed and stained for F-actin. **(F)** Representative fluorescence images showing that recombinant JSH-23 treatment significantly decreased the size of F-actin ring structures. **(G)** Representative SEM and enlarged images of bone resorption pits. **(H)** The bone resorption area was measured relative to that in the control group. The values are shown as the means ± SDs, n = 3; *p < 0.05, **p < 0.01.

The chemical structure of JSH-23 is shown in Figure 1A. To assess the cytotoxicity of the compound, BMMs were exposed to varying doses of JSH-23 for 48 and 96 h. The CCK-8 assay showed that JSH-23 exerted no detectable toxic effects at concentrations under 50 μM (Figure 1B). Therefore, we chose concentrations of 10, 20 and 40 μM JSH-23 for further study. TRAP staining showed that JSH-23 suppressed the total number of cells undergoing osteoclastogenesis and the area of RANKL-induced osteoclastogenesis in a dose-dependent manner (Figures 1C,D). Next, we investigated whether JSH-23 suppressed F-actin formation. F-actin forms loop structures on the bone surface for bone resorption. As shown in Figures 1E,F, phalloidin-Alexa Fluor 488 staining revealed characteristic F-actin rings in osteoclasts without JSH-23 treatment. In contrast, under JSH-23 treatment, the areas of F-actin rings were reduced, and more pleomorphic F-actin rings appeared; these effects were dose-dependent.

Bone resorption is the most important function of osteoclasts and is also one of the critical causes of bone loss. Therefore, we further evaluated whether JSH-23 inhibits osteoclast bone resorption. As shown in Figure 1G, compared to the control treatment, treatment with JSH-23 (10, 20 and 40 μM) reduced the resorption area in a dose-dependent manner (Figure 1H). Collectively, these data confirm that JSH-23 inhibits osteoclastogenesis, F-actin formation and bone resorption with negligible cytotoxicity.

### JSH-23 Downregulates Osteoclast-Specific Gene Expression

**FIGURE 2:**
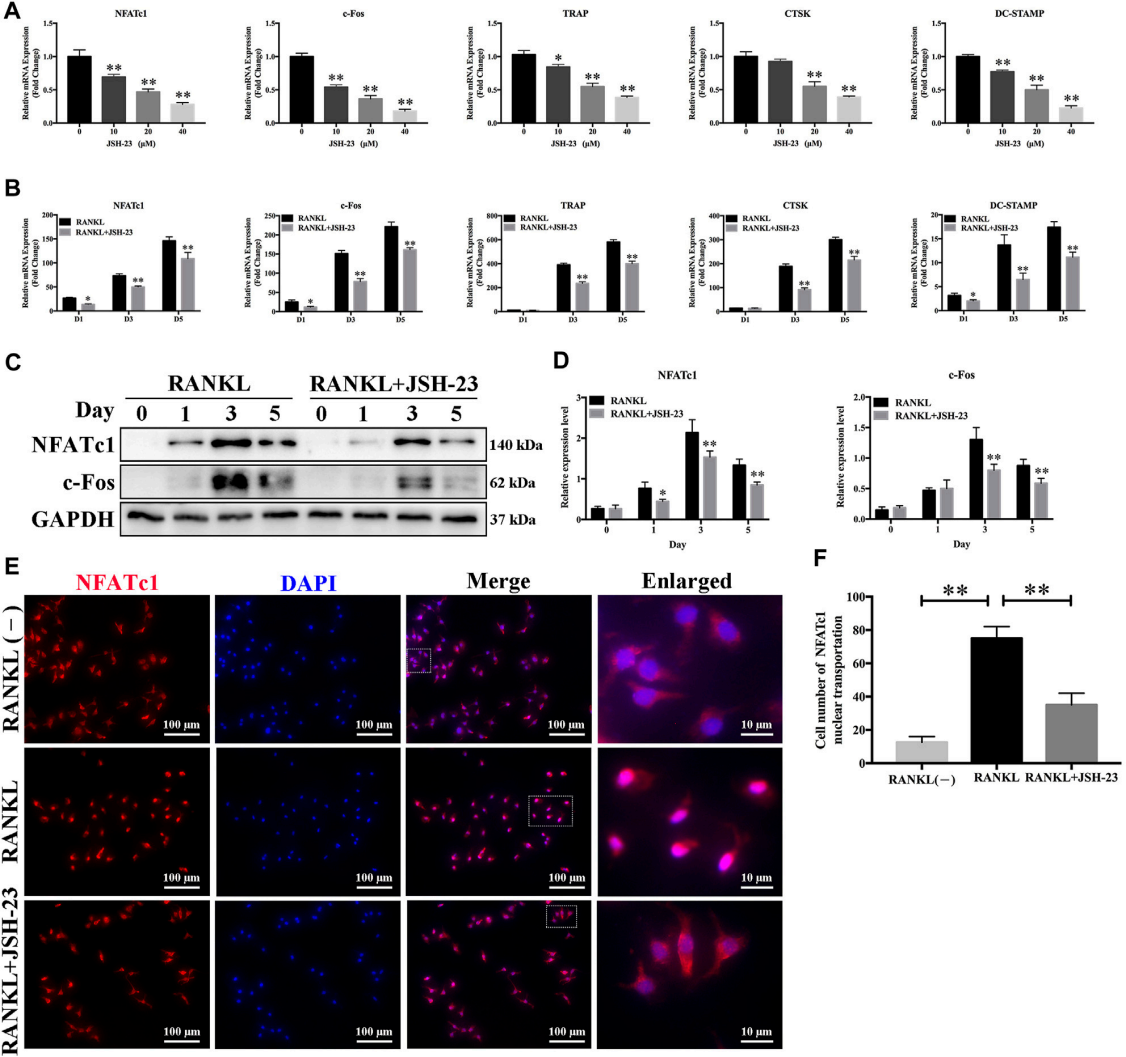
Osteoclast-specific gene expression is inhibited by JSH-23 during osteoclastogenesis. **(A)** BMMs were cultured to generate mature osteoclasts for 5 days with different doses of JSH-23 (10, 20, and 40 μM). **(B)** BMMs were stimulated with M-CSF and RANKL and treated with 40 μM JSH-23 at 1, 3, and 5 days. The mRNA expression levels of NFATc1, c-Fos, TRAP, CTSK, and DC-STAMP were measured by qPCR. **(C)** The protein expression of NfATc1 and c-Fos was analyzed by western blotting (days 1, 3, and 5) and **(D)** quantitatively analyzed. **(E)** Immunofluorescence analysis of BMMs stimulated with RANKL for 3 days to detect the expression of NFATc1. **(F)** Quantitative analysis of cells with NFATc1 nuclear translocation using ImageJ. The values are shown as the means ± SDs, n = 3; *p < 0.05, **p < 0.01.

In order to further clarify the role of JSH-23 in osteoclast differentiation and function, we utilized qPCR to examine the mRNA expression levels of osteoclast-specific genes, including NFATc1, c-Fos, TRAP, CTSK and DC-STAMP. Compared with the control group, the JSH-23-treated groups exhibited strong suppression of RANKL-induced osteoclast-specific gene expression, and this effect of JSH-23 was dose- and time-dependent (Figures 2A,B). Additionally, western blotting assays indicated that this compound inhibited the protein expression of NfATc1 (days 1, 3 and 5) and c-Fos (days 3 and 5) (Figures 2C,D).

During RANKL-induced osteoclastogenesis, NFATc1 self-amplification and nuclear translocation are critical for the expression of osteoclast-specific genes. As expected, NFATc1 nuclear translocation was significantly promoted by RANKL treatment but disrupted by JSH-23 (Figures 2E,F).

These data further demonstrate the inhibitory effects of JSH-23 on osteoclasts.

### JSH-23 Suppresses Osteoclastogenesis via Inhibition of the NF-κB Signaling Pathway

**FIGURE 3:**
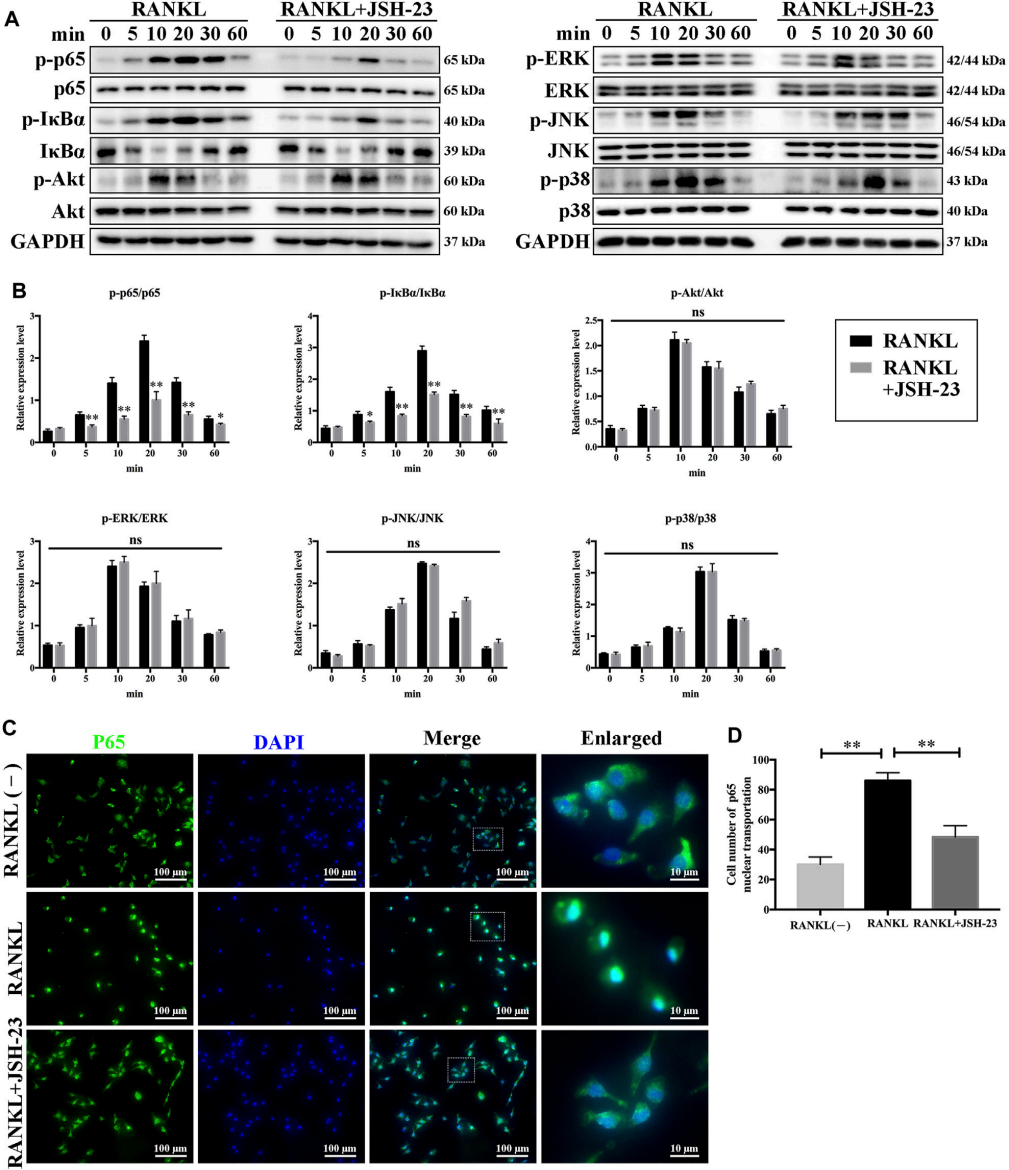
JSH-23 suppresses osteoclastogenesis via inhibition of the NF-κB signaling pathway. **(A)** BMMs were pretreated with JSH-23 (40 μM) for 4 h and thereafter exposed to RANKL (100 ng/ml) for the indicated times (0, 5, 10, 20, 30 and 60 min). The activation of the NF-κB, MAPK and PI3K/Akt signaling pathways was examined by western blotting analysis and **(B)** quantified accordingly. **(C)** BMMs were pretreated with JSH-23 for 4 h and then stimulated with RANKL for 30 min. The representative immunofluorescence images show that recombinant JSH-23 treatment inhibited p65 nuclear transportation. **(D)** Quantitative analysis of cells with p65 nuclear translocation using ImageJ. The values are shown as the means ± SDs, n = 3; ns, no significance; *p < 0.05, **p < 0.01.

To elucidate the molecular mechanisms underlying the effects of JSH-23 on osteoclastogenesis, signaling pathways involved in osteoclastogenesis were investigated. As shown in Figures 3A,B, JSH-23 significantly attenuated p65 and IκBα phosphorylation. However, the phosphorylation levels of Akt, ERK, JNK and p38 were unchanged after JSH-23 treatment. Further immunofluorescence analysis showed that JSH-23 inhibited p65 nuclear translocation (Figure 3C), which was confirmed by quantitative analysis of the number of cells in which p65 was translocated to the nucleus (Figure 3D).

### JSH-23 Suppresses RANKL-Induced ROS Production and Enhances the Nrf2/HO-1 Pathway During Osteoclastogenesis

**FIGURE 4:**
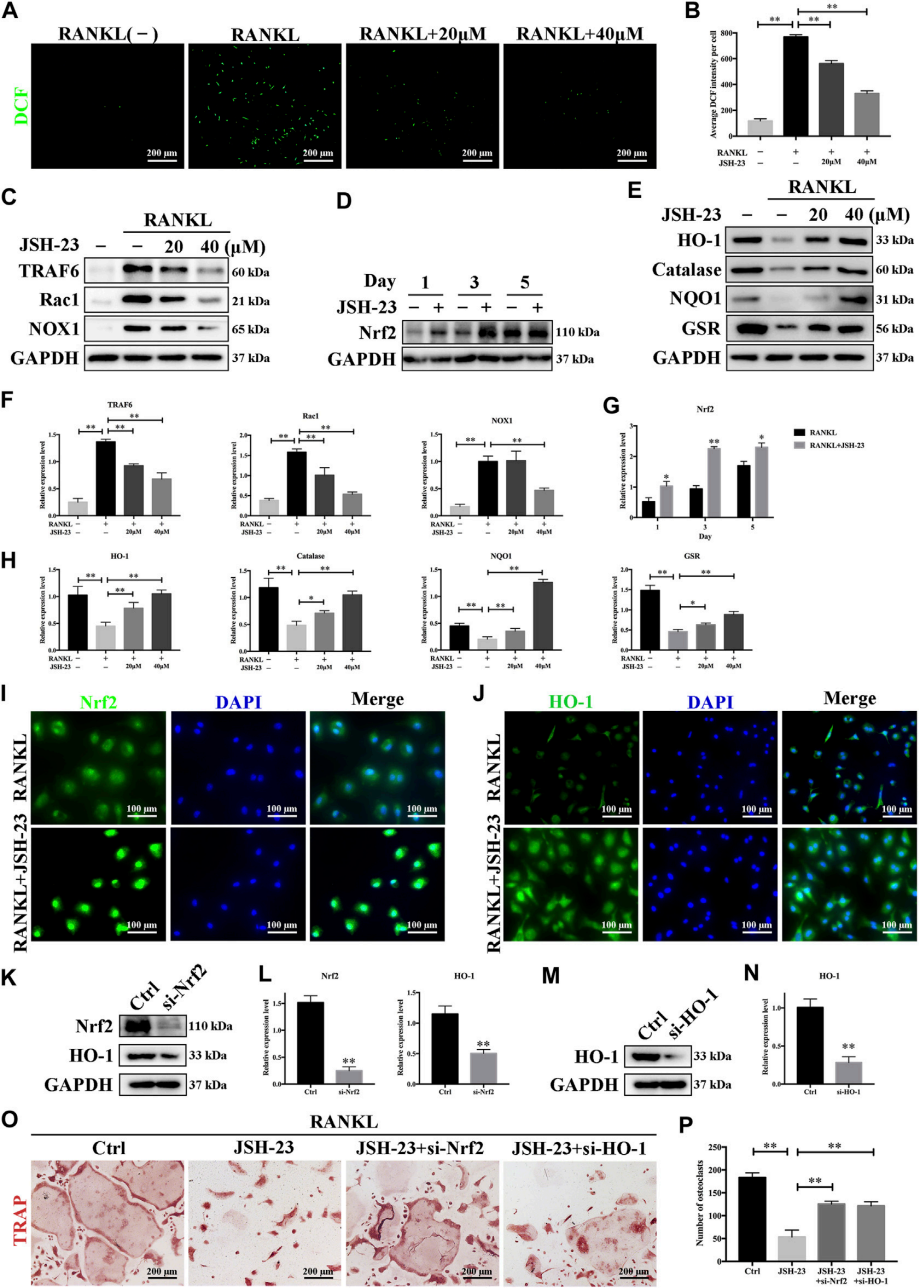
JSH-23 reduces RANKL-induced ROS production during osteoclastogenesis by downregulating the TRAF6/Rac1/NOX1 signaling pathway and enhancing the expression of Nrf2/HO-1. **(A)** Representative images of RANKL-induced ROS generation in BMMs with or without JSH-23 treatment at different concentrations. **(B)** Quantification of the average DCF intensity per well. **(C)** BMMs were stimulated with RANKL in the absence or presence of JSH-23 (20 and 40 μM) for 48 h, and then the ROS production pathway was analyzed by western blotting. **(D)** BMMs were cultured in α-MEM containing M-CSF and RANKL in the presence of JSH-23 (40 μM) at the indicated times (1, 3, 5 days). The expression of Nrf2 was analyzed by western blotting. **(E)** Western blotting images of the effects of JSH-23 on antioxidant enzyme, HO-1, catalase, NQO1, and GSR expression. **(F**–**H)** Quantitative analysis of the western blotting results. **(I)** Representative immunofluorescence images showing Nrf2 translocation and **(J)** HO-1 expression. **(K**–**L)**, BMMs were transfected with siRNA against Nrf2 or **(M, N)** HO-1 for 48 h. The silencing efficiency was evaluated by western blotting. **(O)** Transfected BMMs were treated with RANKL and JSH-23 (40 μM) for 5 days. TRAP staining was measured, and **(P)** the osteoclasts were counted. The values are shown as the means ± SDs, n = 3; *p < 0.05, **p < 0.01.

ROS have been shown to be extremely important in regulating RANKL-dependent osteoclast differentiation and are therefore considered therapeutic targets for the treatment of osteolysis (Chen et al., 2019). As shown in Figure 4A, the intensity of DCF fluorescence was markedly higher in the RANKL-stimulated group than in the control group, but the effect of RANKL stimulation was significantly attenuated in the presence of JSH-23 at concentrations of 20 and 40 μM (Figure 4B). During osteoclastogenesis, RANKL binds to its receptor RANK and generates intracellular ROS through activation of tumor necrosis factor receptor-associated factor 6 (TRAF6), Ras-related C3 botulinum toxin substrate 1 (Rac1), and NADPH oxidase 1 (NOX1). As shown in Figures 4C,F, TRAF6, Rac1 and NOX1 expression was significantly upregulated by RANKL stimulation but dose-dependently inhibited by JSH-23 treatment.

Cells have several protective mechanisms against oxidative stress. Nrf2 is a transcription factor that controls the gene expression of many antioxidant enzymes that combat oxidative stress, such as HO-1, catalase, NAD(P)H:quinone oxidoreductase 1 (NQO1), and glutathione reductase (GSR) (Kleszczynski et al., 2016). We demonstrated that JSH-23 activated Nrf2 expression during RANKL-induced osteoclastogenesis (Figures 4D,G). HO-1, one of the main oxidative stress markers induced by Nrf2 activation, is involved in defense against various oxidative stress-inducing agents. The expression of HO-1 was reduced by RANKL stimulation but was recovered and enhanced dose-dependently by JSH-23 treatment. Similarly, JSH-23 enhanced the expression levels of catalase, NQO1 and GSR (Figures 4E,H).

Nrf2 nuclear translocation is a prerequisite for activation of downstream antioxidative target genes. Immunofluorescence staining for Nrf2 and nuclei (DAPI) in BMMs revealed that Nrf2 was localized evenly in the cytoplasm of control cells, while the intensity of green staining was markedly increased in nuclei or concentrated on the edge of nuclei in cells treated with JSH-23 (Figure 4I). In addition, JSH-23 increased HO-1 fluorescence intensity (Figure 4J), consistent with our western blotting results.

Next, to confirm that the Nrf2/HO-1 antioxidant pathway is involved in the inhibition of osteoclastogenesis by JSH-23, we silenced Nrf2 or HO-1 expression using small interfering RNA (siRNA), and the silencing effect was confirmed by western blotting analysis (Figures 4K–N). Interestingly, silencing Nrf2 reduced HO-1 expression, further indicating that HO-1 is downstream of Nrf2 (Figure 4K). Moreover, downregulation of Nrf2 or HO-1 attenuated the inhibitory effect of JSH-23 (Figure 4O), reversed the impairment of osteoclastogenesis, and caused mature osteoclasts to appear (Figure 4P). Collectively, these results indicate that JSH-23 reduces RANKL-induced ROS production in osteoclastogenesis by downregulating the TRAF6/Rac1/NOX1 signaling pathway and enhancing the expression of Nrf2/HO-1.

### JSH-23 Attenuates the Apoptotic Effect of H_2_O_2_ in MC3T3-E1 Cells

**FIGURE 5:**
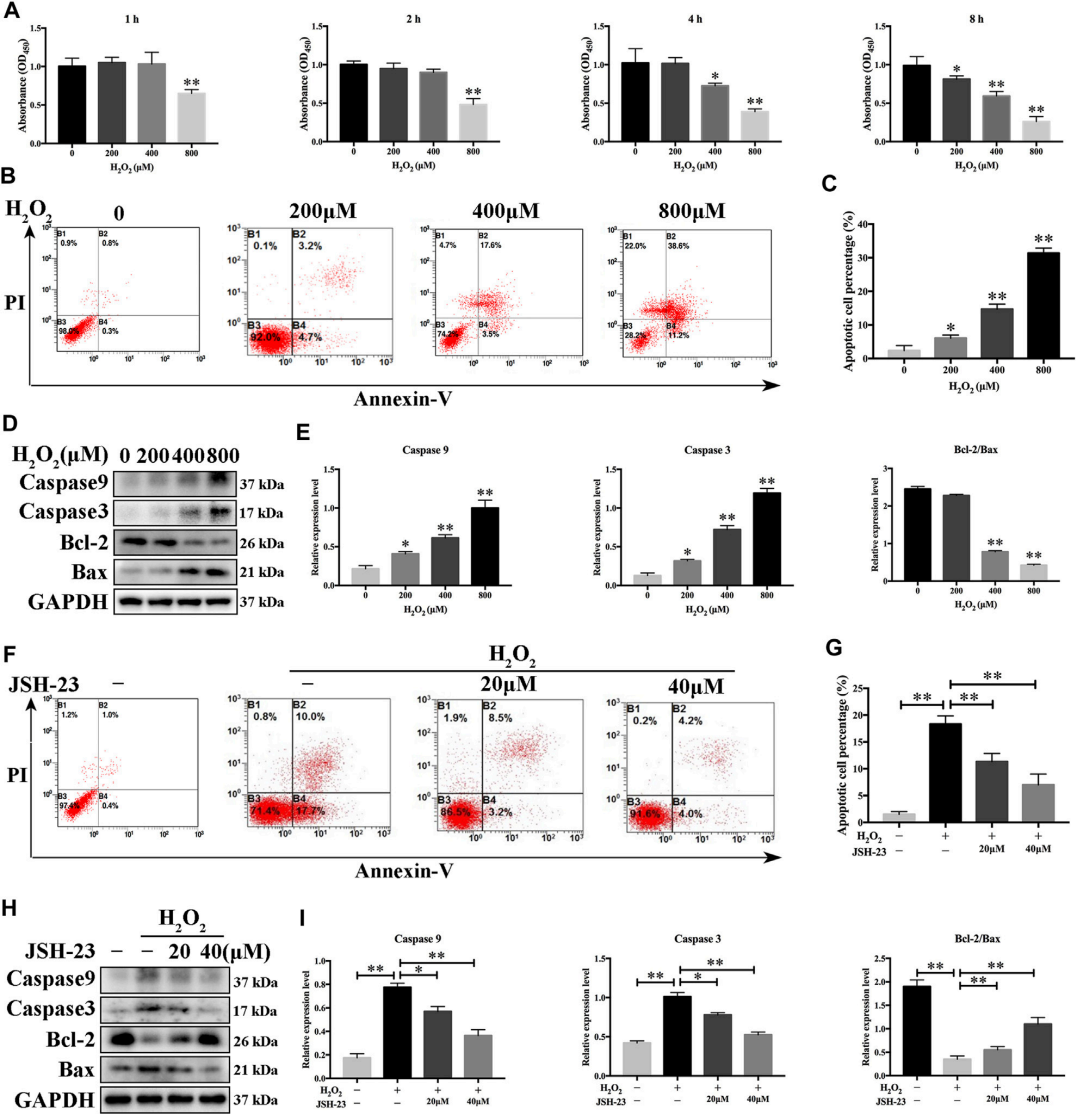
Inhibitory effects of JSH-23 on H_2_O_2_-induced apoptosis in MC3T3-E1 cells. **(A)** MC3T3-E1 cells were treated with various concentrations of H_2_O_2_ (0–800 μM) for 1, 2, 4 and 8 h, and cell viability was analyzed by CCK-8 assay. (B) Cells were treated with 0, 200, 400 and 800 μM H_2_O_2_ for 4 h, and apoptosis was determined by flow cytometry followed by FITC-Annexin V and PI double staining. **(C)** The percentage of apoptotic cells in each well was quantified. **(D**–**E)** The protein levels of cleaved Caspase-9, cleaved Caspase-3, Bcl-2 and Bax were detected by western blotting. **(F)** MC3T3-E1 cells were pretreated with JSH-23 (40 μM) for 24 h and then treated with H_2_O_2_ (400 μM) for 4 h. Apoptosis was measured by flow cytometry followed by FITC-Annexin V and PI double staining. **(G)** The percentage of apoptosis was determined. **(H)** Cells were treated for 4 h with H_2_O_2_ in the presence or absence of JSH-23. Western blotting analysis was performed using the indicated antibodies. **(I)** Quantitative analysis of the western blotting results. The values are shown as the means ± SDs, n = 3; *p < 0.05, **p < 0.01.

Since osteoclasts and osteoblasts are both important in bone homeostasis, the effects of JSH-23 on osteoblasts were studied. Although ROS maintain osteoclast differentiation and survival, oxidative stress induces osteoblast apoptosis, reduces osteogenic bone formation, decreases bone mass and triggers bone loss (Tao et al., 2020). Here, we found that the viability of osteoblasts decreased in a dose- and time-dependent manner when the cells were exposed to different concentrations of H_2_O_2_ (range of 0–800 μM) (Figure 5A). In addition, flow cytometric analysis demonstrated that osteoblast apoptosis increased when cells were exposed to H_2_O_2_ (Figures 5B,C). According to the results, 400 μM H_2_O_2_ for 4 h was chosen as the condition for oxidative stress model establishment in MC3T3-E1 cells for further research. Western blotting analysis showed that H_2_O_2_ increased cleaved caspase-9 and cleaved caspase-3 expression and decreased the Bcl-2/Bax ratio in a dose-dependent manner (Figures 5D,E). These data confirmed that H_2_O_2_ induced apoptosis in MC3T3-E1 cells. Notably, treatment with JSH-23 decreased the percentage of apoptotic cells under H_2_O_2_ treatment (Figures 5F,G). Further research showed that JSH-23 exerted its antiapoptotic effect by decreasing cleaved caspase-9 and cleaved caspase-3 expression and increasing the Bcl-2/Bax ratio (Figures 5H,I).

### JSH-23 Prevents H_2_O_2_-Induced Disruption of Osteoblast Function

**FIGURE 6:**
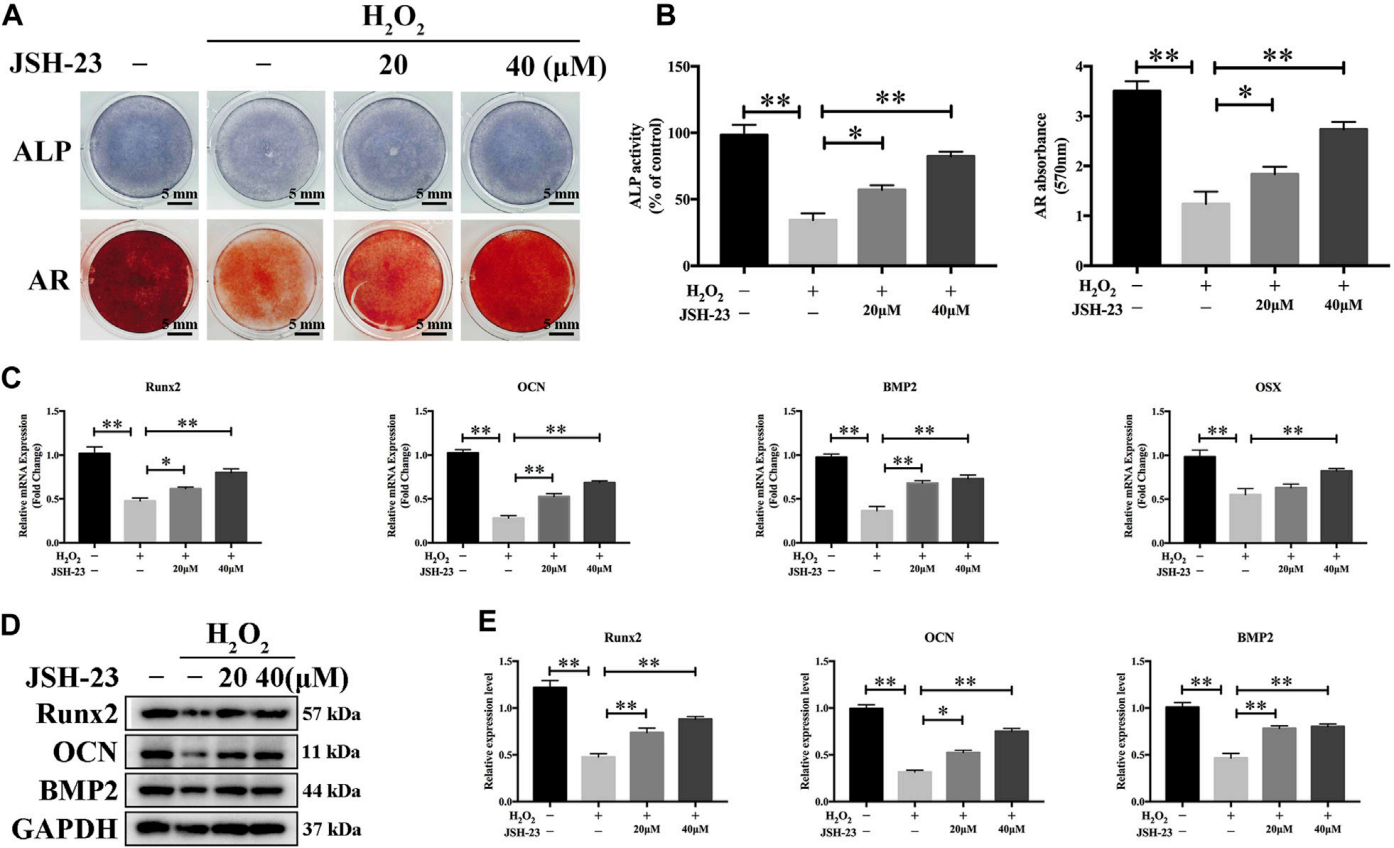
JSH-23 alleviates H_2_O_2_-induced inhibition of osteoblast differentiation in MC3T3-E1 cells. **(A)** Cells were pretreated with JSH-23 for 24 h before induction of differentiation until day 7 or 21 and then exposed to H_2_O_2_ (400 μM) for 4 h. ALP or AR staining was performed. **(B)** Quantitative analysis of ALP activity and AR staining in cells based on absorbance. **(C)** The expression of the osteoblast-specific genes (Runx2, OCN, BMP2 and OSX) was analyzed by qPCR. **(D)** The protein expression levels of Runx2, OCN and BMP2 were measured using western blotting. **(E)** Quantitative analysis of the western blotting results. The values are shown as the means ± SDs, n = 3; *p < 0.05, **p < 0.01.

We further evaluated the effect of JSH-23 on H_2_O_2_-induced changes in osteoblast function during early differentiation and late-stage mineralization *in vitro*. We found that H_2_O_2_ markedly reduced osteoblast differentiation, as measured by analysis of ALP staining at 7 days and AR staining at 21 days (Figures 6A,B). However, pretreatment with JSH-23 for 24 h markedly alleviated the subsequent H_2_O_2_-mediated downregulation of ALP expression and reduction in mineralization (Figures 6A,B).

To investigate the effects of JSH-23 on H_2_O_2_-induced osteogenic dysfunction, we investigated the mRNA and protein expression levels of osteoblast markers by qPCR and western blotting analysis, respectively. H_2_O_2_ markedly downregulated the mRNA levels of Runx2, OCN, BMP2 and OSX, while pretreatment with 20 and 40 μM JSH-23 increased the mRNA levels of these genes (Figure 6C). Similar trends were observed at the protein level: JSH-23 pretreatment prevented H_2_O_2_-induced marked reductions in Runx2, OCN and BMP2 levels (Figures 6D,E). Collectively, these results suggest that JSH-23 pretreatment prevents H_2_O_2_-induced decreases in osteogenic differentiation.

### JSH-23 Scavenges Intracellular ROS Through Increased Activation of the Nrf2/HO-1 Pathway in MC3T3-E1 Cells

**FIGURE 7:**
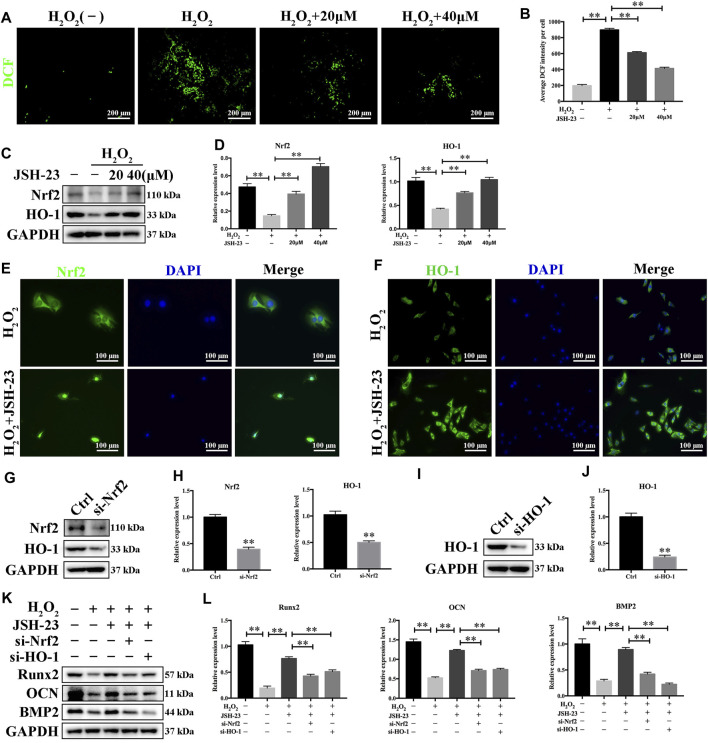
JSH-23 scavenges intracellular ROS through increased activation of the Nrf2/HO-1 pathway in MC3T3-E1 cells. **(A)** MC3T3-E1 cells were pretreated with JSH-23 (20 and 40 μM) for 24 h before stimulation with H_2_O_2_ (400 μM), and ROS generation was observed using fluorescence microscopy. **(B)** The fluorescence intensity of ROS was measured with ImageJ software. **(C)** Nrf2 and HO-1 expression was determined by western blotting analysis. **(D)** Quantitative analysis of the western blotting results. **(E)** MC3T3-E1 cells were pretreated with JSH-23 (20 and 40 μM) for 24 h before stimulation with H_2_O_2_ (400 μM). The representative immunofluorescence images show Nrf2 translocation and **(F)** HO-1 expression. **(G**–**H)** MC3T3-E1 cells were transfected with siRNA against Nrf2 or **(I, J)** HO-1 for 48 h. The transfection efficiency was confirmed by western blotting. **(K)** The protective effects of JSH-23 on the expression of the bone markers (Runx2, OCN and BMP2) in H_2_O_2_-treated MC3T3-E1 cells were greatly weakened by Nrf2 or HO-1 knockdown. **(L)** Quantitative analysis of the western blotting results. The values are shown as the means ± SDs, n = 3; **p < 0.01.

Treatment of MC3T3-E1 cells with H_2_O_2_ alone clearly increased the levels of ROS. However, pretreatment with JSH-23 significantly prevented this effect (Figures 7A,B). Western blotting analysis demonstrated that 20 and 40 μM JSH-23 treatment significantly increased Nrf2 and HO-1 protein levels (Figures 7C,D). Similar to the results showing that JSH-23 promoted the Nrf2/HO-1 pathway in osteoclasts, the immunofluorescence analysis results showed that JSH-23 promoted Nrf2 nuclear translocation and increased HO-1 fluorescence intensity in MC3T3-E1 cells (Figures 7E,F).

Next, we silenced Nrf2 and HO-1 expression using siRNA in MC3T3-E1 cells, and the silencing effect was confirmed by western blotting (Figures 7G–J). After downregulation of Nrf2 or HO-1, the protective effect of JSH-23 against the H_2_O_2_-induced reductions in the levels of osteoblast markers (Runx2, OCN and BMP2) was attenuated (Figures 7K,L). These results suggest that JSH-23 prevents H_2_O_2_-induced impairment of osteoblast differentiation by scavenging intracellular ROS and increasing activation of the Nrf2/HO-1 pathway.

### JSH-23 Prevents LPS-Induced Osteolysis *in vivo*

**FIGURE 8:**
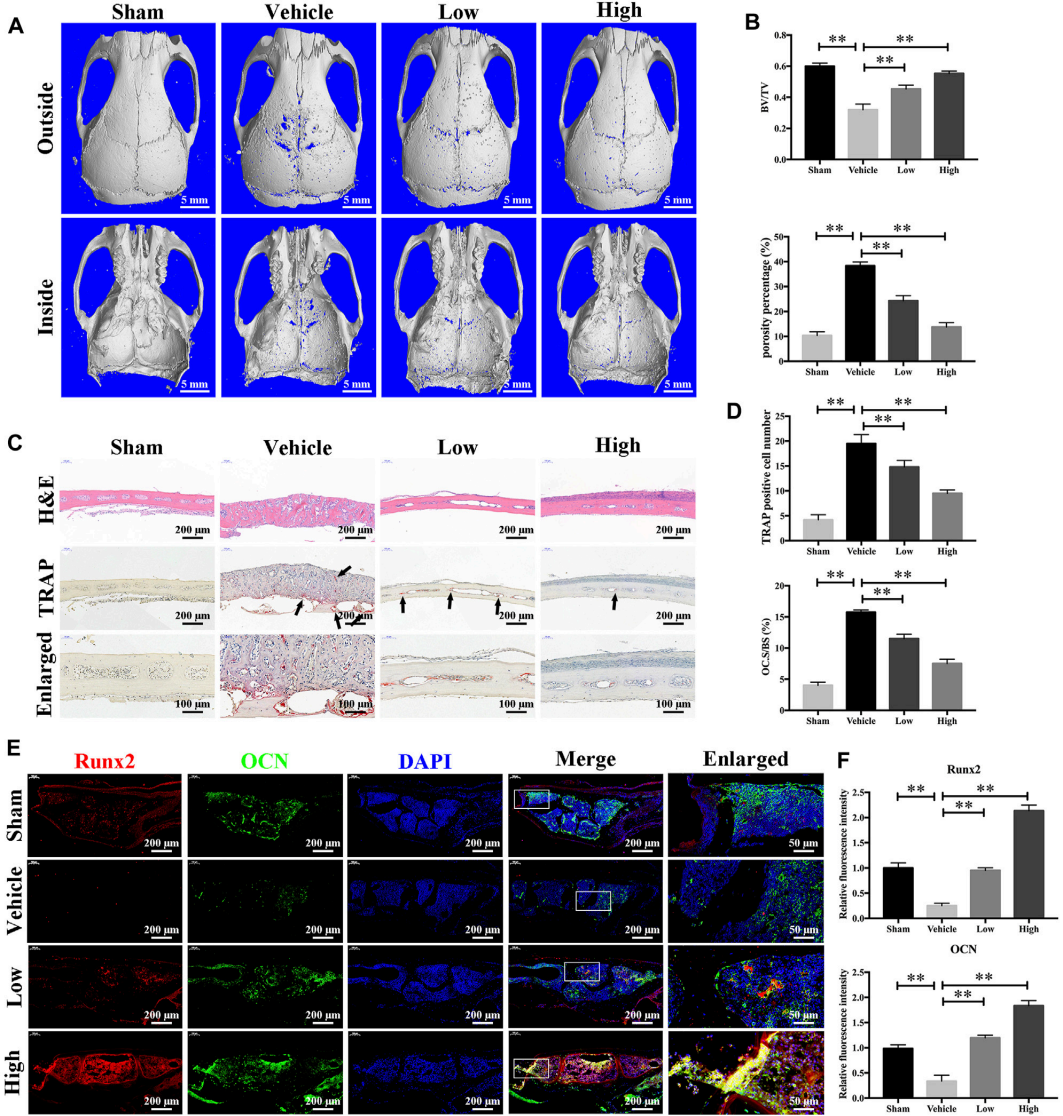
JSH-23 prevents LPS-induced osteolysis *in vivo*. **(A)** Micro-CT scanning and 3D reconstruction of whole calvaria from the sham group (PBS), vehicle group (LPS), low group (LPS with 1 mg/kg JSH-23) and high group (LPS with 3 mg/kg JSH-23). **(B)** Quantitative analysis of the bone volume/total volume (BV/TV) ratio and porosity percentage. **(C)** Representative images of calvaria stained with H&E and TRAP from each group. **(D)** The total TRAP-positive cell number and the OC.S/BS percentage were quantified. **(E)** Immunofluorescence analysis of the expression of Runx2 and OCN in calvarial tissue sections from each group. **(F)** Quantitative analysis of the relative fluorescence intensity of Runx2 and OCN in each experimental group. The values are shown as the means ± SDs, n = 6; **p < 0.01.

Having determined that JSH-23 inhibits osteoclastogenesis and promotes osteoblastogenesis *in vitro*, we next explored the potential protective effect of JSH-23 under the pathologic setting of LPS-induced osteolysis *in vivo*. As shown in the three-dimensional reconstruction images in Figure 8A, compared to the sham group, the vehicle group exhibited extensive erosion on the calvarial bone surface. However, the severity of LPS-induced osteolysis was significantly inhibited by JSH-23 treatment (Figure 8B). Consistent with the micro-CT results, H&E staining showed that JSH-23 significantly reduced the area of LPS-induced bone erosion (Figure 8C). TRAP staining further revealed that JSH-23 treatment dose-dependently decreased the numbers of TRAP-positive osteoclasts and osteoclasts lining the bone surface (OC.S/BS) (Figures 8C,D).

The anabolic effect of JSH-23 on bone formation *in vivo* was also examined. Calvarial bone sections were subjected to immunofluorescence double staining for the osteoblast-specific markers Runx2 and OCN. As shown in Figure 8E, the JSH-23-treated group exhibited higher expression of Runx2 and OCN on the calvarial bone surface than the vehicle group (Figure 8F). Taken together, these results provide evidence that JSH-23 is an effective agent for the treatment of pathologic bone loss conditions, such as LPS-induced osteolysis.

### JSH-23 Reduces ROS Production and Enhances Nrf2/HO-1 Expression *in vivo*

**FIGURE 9:**
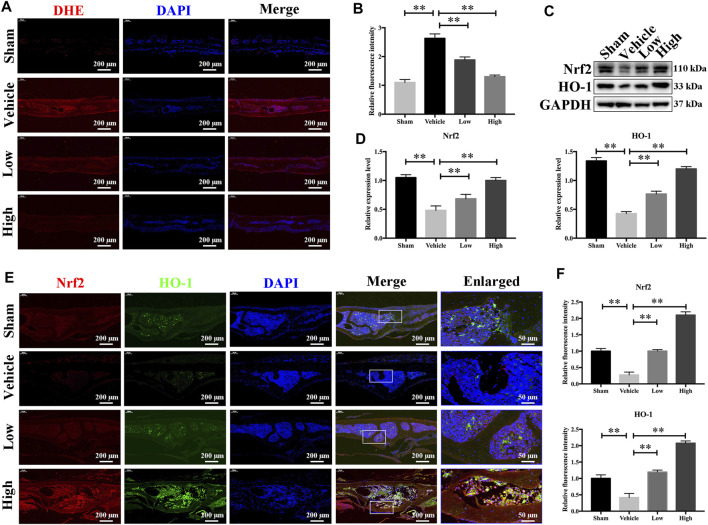
JSH-23 reduces ROS production and enhances the expression of Nrf2/HO-1 *in vivo*. **(A)** Representative images of bone sections showing DHE fluorescence in different groups. **(B)** Quantification of the DHE fluorescence intensity of each group. **(C)** Proteins were isolated from bone tissues, and Nrf2/HO-1 expression was investigated using western blotting. **(D)** Quantitative analysis of the western blotting results. **(E)** Immunofluorescence double staining was performed to detect the expression of Nrf2 and HO-1. **(F)** Quantitative analysis of Nrf2 and HO-1 immunofluorescence intensity. The values are shown as the means ± SDs, n = 6; **p < 0.01.

**FIGURE 10:**
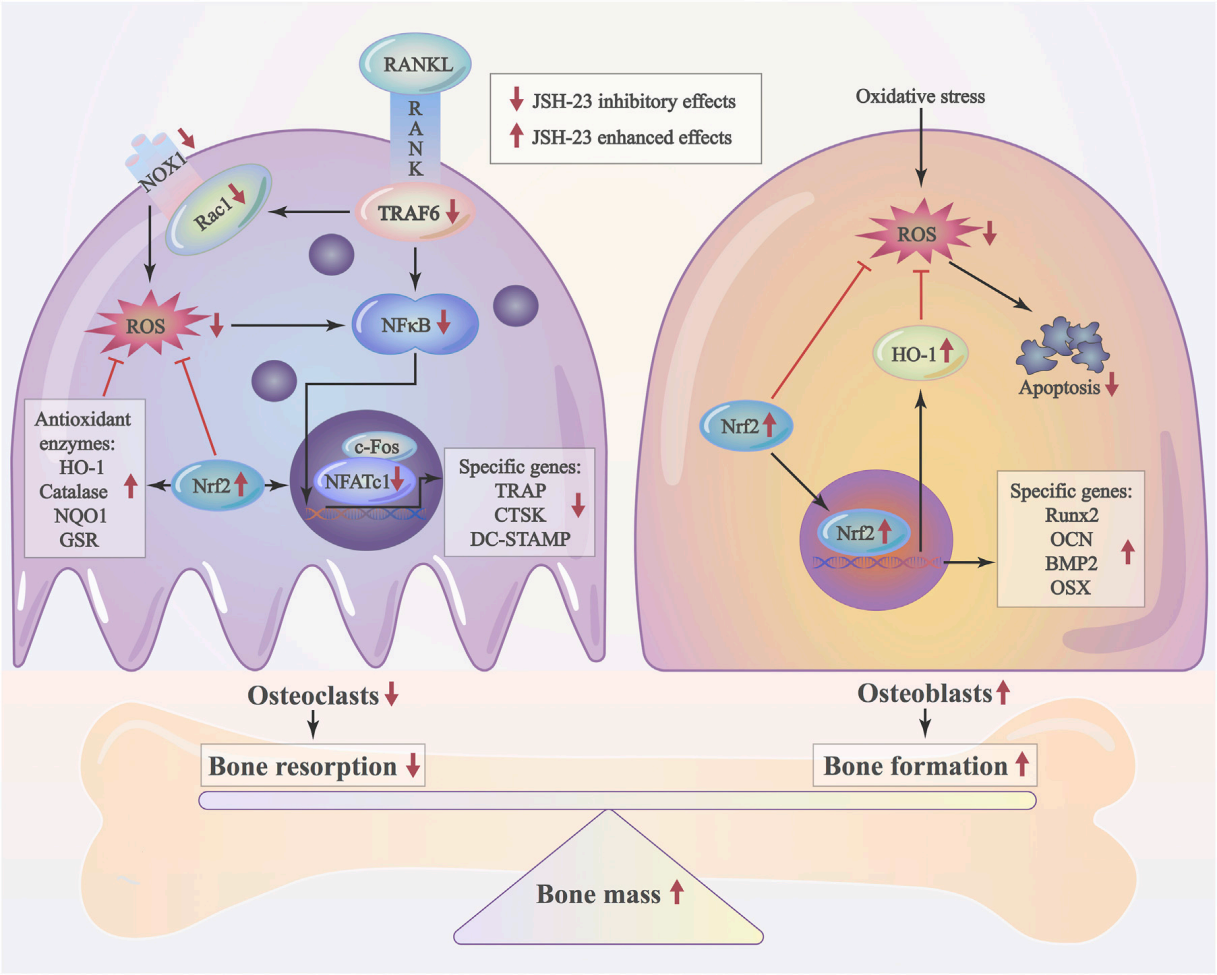
Schematic diagram of the possible mechanism by which JSH-23 elevates bone mass by scavenging ROS production and activating Nrf2/HO-1 signaling.

As JSH-23 exhibited intracellular antioxidant activity, we next assessed *in vivo* ROS levels on the bone surface using the ROS probe DHE. Consistent with the *in vitro* results, ROS fluorescence intensity in bone tissue was much higher in the vehicle group than in the sham group, but JSH-23 dramatically reversed ROS production *in vivo* (Figures 9A,B). To investigate Nrf2 and HO-1 expression *in vivo*, total protein was extracted from each group and detected using western blotting. Enhancement of Nrf2 and HO-1 expression was observed in the JSH-23-treated group (Figures 9C,D). Furthermore, to confirm our western blotting results, immunofluorescence double staining for Nrf2 and HO-1 were performed. The results showed that Nrf2 and HO-1 staining were more intense in the JSH-23-treated group than in the vehicle group (Figures 9E,F). A proposed scheme of the mechanism by which JSH-23 restrains bone loss by scavenging ROS production and activating Nrf2/HO-1 signaling is shown in Figure 10. Collectively, these results demonstrate that JSH-23 prevents LPS-induced osteolysis *in vivo* by reducing ROS production and enhancing Nrf2/HO-1 expression.

## Discussion

Osteolysis is a pathological skeletal disease that is characterized by low bone mass and is associated with high morbidity, mortality, and healthcare costs (Mbalaviele et al., 2017). However, long-term preventive and curative medical care is not currently available. Emerging studies have suggested that ROS play a critical role in bone homeostasis (Chen et al., 2019; Xu et al., 2019; Ni et al., 2020). However, excessive ROS generation and oxidative status contribute to the progression of bone destruction (Tao et al., 2020). 4-Methyl-N^1^-(3-phenyl-propyl)-benzene-1,2-diamine (JSH-23) is a novel chemically synthetic compound with transcriptional inhibitory activity by targeting NF-κB. The aromatic diamine JSH-23 compound exhibited inhibitory effect with an IC50 value of 7.1 μM on NF-κB transcriptional activity in LPS-stimulated RAW 264.7 macrophages, and interfered LPS-induced nuclear translocation of NF-κB without affecting IκB degradation. This mechanism of action is very rare for controlling NF-κB activation (Shin et al., 2004).

As an aromatic diamine compound (Figure 1A), JSH-23 may not be able to directly scavenge ROS by depending on the galloyl group and the phenolic hydroxyl group, as tea polyphenols do (Mao et al., 2019). However, previous studies have demonstrated that JSH-23 could indirectly eliminate ROS through improving anti-oxidative enzymes activities and decreasing the inflammatory reaction, which involves the regulation of Nrf2 and NF-κB signaling pathways (Kumar et al., 2011; Wang et al., 2018). For example, in the chronic mild stress mouse model, administration of JSH-23 significantly prevented depressive-like behaviors by decreasing inflammation (p65, IL-6 and TNF-α) and improving antioxidant defense (SOD and Nrf2) in the hippocampus (Wang et al., 2018). Another study indicates that inhibition of NF-κB inflammatory cascade using JSH-23 reversed the functional, sensorimotor and biochemical deficits by decreasing neuroinflammation (IL-6, TNF-α, COX-2 and iNOS) and improving antioxidant defence (Nrf2 and HO-1) in diabetic neuropathy (Kumar et al., 2011). In our study, JSH-23 can inhibit osteoclastogenesis and protect osteoblasts by enhancing the expression of members of the antioxidant Nrf2/HO-1 pathway *in vitro* and *in vivo*. Mechanistically, JSH-23 suppresses RANKL-induced osteoclastogenesis, bone resorption and the expression of specific genes (including NFATc1, c-Fos, TRAP, CTSK and DC-STAMP) via inhibition of the NF-κB signaling pathway. Meanwhile, JSH-23 suppresses RANKL-induced ROS production via the Nrf2/HO-1 pathway during osteoclastogenesis. In addition, JSH-23 attenuates H_2_O_2_-induced apoptosis and mineralization reduction in osteoblasts by reducing ROS production and enhancing the Nrf2/HO-1 pathway *in vitro*. Our *in vivo* results further reveal the antioxidation-mediated protective effect of JSH-23 on bone mass. Given these findings, application of JSH-23 might be a novel and plausible strategy for the treatment of osteolysis-related disease.

Osteoclasts are usually differentiated from the monocyte/macrophage lineage upon stimulation with macrophage colony-stimulating factor (M-CSF) and receptor activator of nuclear factor-κB ligand (RANKL)(Hedvicakova et al., 2021). RANKL is an essential cytokine during formation and activation of osteoclasts. After RANKL binds to RANK, it will recruit TRAF6 and then activates downstream NF-κB signal pathway, followed by the activation of transcription factors like activator protein 1 (AP-1) and nuclear factor of activated T-cell cytoplasmic 1 (NFATc1). Finally, NFATc1 co-operates with AP-1 and other factors to produces osteoclast-specific genes which leads to the formation and survival of osteoclasts, and bone resorption (Park et al., 2017; Kitazawa et al., 2021). In our *in vitro* model, JSH-23 dose-dependently inhibited RANKL-induced osteoclast differentiation and bone resorption (Figure 1). JSH-23 downregulated master regulators of osteoclast differentiation, including NFATc1 and other related genes (Figure 2). JSH-23 inhibited p65 phosphorylation in our study (Figure 3A), which suggests that JSH-23 suppresses the nuclear translocation of p65. This hypothesis was supported by the results of immunofluorescence staining (Figures 3C,D). The results reveal that RANKL-induced NF-κB signaling is involved in the inhibitory effect of JSH-23 on osteoclastogenesis.

LPS has long been recognized as a major mediator of bone loss in chronic infection by Gram-negative bacteria (Mormann et al., 2008). Injection of LPS in animals results in severe bone resorption (Kim et al., 2021). Different to RANKL signaling, LPS-induced activation of osteoclasts is mainly mediated by TLR4 transmembrane protein (AlQranei et al., 2021). Binding with its receptor TLR4, LPS also recruits TRAF6 into cytoplasm and further activates NF-κB signaling pathway (He et al., 2016). In LPS-induced osteoclastogenesis, increases in expression of TLR4 and TRAF6 as well as downstream NF-κB, and some osteoclast relative genes and cytokines, have been documented (He et al., 2016; Zeng et al., 2020). In our LPS-induced osteolysis animal model, LPS injection group exhibited extensive erosion on the calvarial bone surface (Figures 8A,B). After JSH-23 treatment, the severity of LPS-induced osteolysis was significantly decreased (Figures 8A–C), the numbers of TRAP-positive osteoclasts and osteoclasts lining the bone surface were attenuated (Figures 8C,D). As TLR4 is the membrane receptor of LPS and play an essential role in LPS-induced osteoclastogenesis, our *in vivo* results suggest that JH-23 may have an inhibitory effect on TLR4 pathway. Therefore, the mechanisms of JSH-23 on LPS/TLR4 pathway in osteoclasts will be investigated in the future.

ROS are natural byproducts of the normal metabolism of oxygen. However, when their concentrations exceed the normal range, oxidative stress can disrupt the balance between oxidation and antioxidant defense systems; such disruption can reduce the levels of antioxidant enzymes, inducing osteoblast apoptosis but promoting bone resorption by osteoclasts (Zhang et al., 2016; Tao et al., 2020). This disturbance can lead to various osteolytic diseases, such as periodontitis, rheumatoid arthritis and osteoporosis (Wruck et al., 2011; Chen et al., 2019). Cells have several protective mechanisms against this oxidative stress [8]. One of the major factors is the transcription factor Nrf2, which binds to antioxidant response elements (AREs) and controls the gene expression of many antioxidant enzymes, such as HO-1, catalase, NQO1 and GSR (Kanzaki et al., 2016). HO-1-dependent signaling is an important adaptive mechanism for homeostasis maintenance because of the anti-inflammatory and antiapoptotic properties of HO-1 and augments cellular resistance to oxidative stress (Zhang et al., 2002; Abraham and Kappas, 2008). Induction of HO-1 with transient gene vectors or natural bioactive components inhibits osteoclastogenesis (Fumimoto et al., 2012; Sakai et al., 2012). In contrast, downregulation of HO-1 via RNA interference enhances osteoclast formation (Sakai et al., 2012). Furthermore, HO-1 has been suggested to be a mediator of *in vitro* osteoblastogenesis in primary rat and human MSCs treated with osteoprotegerin (Barbagallo et al., 2010; Kozakowska et al., 2014). In our study, HO-1 participated in both osteoclastogenesis and osteoblastogenesis, and downregulation of HO-1 expression contributed to enhanced osteoclastogenesis (Figure 4O) and reductions in osteoblast-specific marker expression (Figure 7K). These lines of evidence suggest that agents that induce HO-1 may exert protective effects against diseases involving bone loss.

Many studies have shown that global Nrf2 knockout increases intracellular ROS, activation of RANKL-induced pathways and osteoclastic gene expression through downregulation of the antioxidant response (Wruck et al., 2011; Hyeon et al., 2013; Ibanez et al., 2014), while overexpression of Nrf2 inhibits osteoclastogenesis (Kanzaki et al., 2013), making Nrf2 a promising molecular drug target for osteoclast-related diseases. Our results revealed that knockdown of Nrf2 with siRNA reversed the inhibitory effect of JSH-23 in osteoclasts, further illustrating the critical role of Nrf2 in osteoclastogenesis. Nevertheless, the effects of Nrf2 on osteoblast differentiation are controversial. A previous study has reported that Nrf2 negatively regulates osteoblast differentiation (Hinoi et al., 2006). Nrf2-knockout mice present increased mineral apposition rates and osteoblast numbers (Park et al., 2014). Other studies have suggested that Nrf2-knockout mice have impaired bone metabolism and diminished load-driven bone formation (Sun et al., 2015) and exhibit marked deficits in bone acquisition since early osteoblastogenesis in bone marrow stromal cells (BMSCs) is impaired by increased reactive species-related stress (Kim et al., 2014). Nrf2 knockdown observably abrogates the beneficial effect of Z-Guggulsterone in MC3T3-E1 cells (Xu et al., 2019). Consistent with this finding, our research revealed that JSH-23 attenuated H_2_O_2_-induced apoptosis and mineralization reduction by activating Nrf2 expression *in vitro* and *in vivo*. Further detailed studies are necessary to explore the effects of JSH-23 on Nrf2 activation in osteoblasts.

As the name of RANKL (receptor activator of NF-κB ligand) implies, NF-κB signaling is mediated by RANKL during osteoclastogenesis. In its resting state, NF-κB is present in the cytoplasm and bound in an inactive form to the IκBα inhibitory protein. However, when IκBα is phosphorylated by the IκB kinase complex upon binding of RANKL to RANK, the released NF-κB p65 translocates from the cytoplasm to the nucleus and triggers transcriptional activation of several osteoclast-related genes. Our western blotting and immunofluorescence staining results indicated that JSH-23 inhibited RANKL-induced p-p65 expression and nuclear translocation, respectively. Recently, several studies have reported that ROS can stimulate the NF-κB signaling pathway in testicular cells (Guo et al., 2017), alveolar epithelial cells (Lin et al., 2016), fibroblasts and other cell types (Shin et al., 2017). Accumulating evidence indicates that ROS may also influence the activity of NF-κB, a key osteoclast transcription factor, by disrupting the phosphorylation of IκBα (Ha et al., 2004). In turn, NF-κB can regulate ROS activity by enhancing the production of antioxidant enzymes (Zhang et al., 2016). Therefore, NF-κB signaling might be downstream of RANKL-mediated ROS signaling, or ROS signaling might be the modulator of NF-κB signaling. Further studies are needed to investigate the mechanisms of JSH-23 in ROS-NF-κB crosstalk in osteoclasts. We are very interested in researching this topic in the future.

In conclusion, our findings demonstrate that JSH-23 restrains bone loss by reducing ROS production and activating the Nrf2/HO-1 pathway. Targeting Nrf2/HO-1 with JSH-23 may be a promising therapeutic strategy for overcoming osteolysis.

## Data Availability

The original contributions presented in the study are included in the article/Supplementary Material, further inquiries can be directed to the corresponding authors.
