# Supplementary material for: The Novel Antioxidant Compound JSH-23 Prevents Osteolysis by Scavenging ROS During Both Osteoclastogenesis and Osteoblastogenesis
Source: Front Pharmacol. 2021 Sep 9;12:734774. doi: 10.3389/fphar.2021.734774 (PMC8458573; doi:10.3389/fphar.2021.734774)
Supplement: Supplementary file 1 [file Table1.DOCX]

Table S1. The mouse primer sequences used in real-time PCR.

| Gene | Sequence (5′-3′) | |
| --- | --- | --- |
| NFATc1 | Forward | TCCACCCACTTCTGACTTCC |
|  | Reverse | CTTCGCCCACTGATACGAG |
| DC-STAMP | Forward | GCTGTATCGGCTCATCTCCT |
|  | Reverse | AAGGCAGAATCATGGACGAC |
| c-Fos | Forward | GTTCGTGAAACACACCAGGC |
|  | Reverse | GGCCTTGACTCACATGCTCT |
| CTSK | Forward | TCCGCAATCCTTACCGAATA |
|  | Reverse | AACTTGAACACCCACATCCTG |
| TRAP | Forward | CCATTGTTAGCCACATACGG |
|  | Reverse | CACTCAGCACATAGCCCACA |
| BMP2 | Forward | GATCTGTACCGCAGGCACTC |
|  | Reverse | TTCCCACTCATCTCTGGAAGTT |
| Runx2 | Forward | AGATGACATCCCCATCCATC |
|  | Reverse | GTGAGGGATGAAATGCTTGG |
| OCN | Forward | GGCGTCCTGGAAGCCAATGTG |
|  | Reverse | GACCAGGAGGACCAGGAAGTCCACGT |
| OSX | Forward | CTTCCCAATCCTATTTGCCGTTT |
|  | Reverse | CGGCCAGGTTACTAACACCAATCT |
| GAPDH | Forward | ACCCAGAAGACTGTGGATGG |
|  | Reverse | CACATTGGGGGTAGGAACAC |
